# Supplementary material for: Differential dynamics of cortical neuron dendritic trees revealed by long-term in vivo imaging in neonates
Source: Nat Commun. 2018 Aug 6;9:3106. doi: 10.1038/s41467-018-05563-0 (PMC6078955; doi:10.1038/s41467-018-05563-0)
Supplement: Supplementary file 1 — Supplementary Information [file 41467_2018_5563_MOESM1_ESM.pdf]

Differential dynamics of cortical neuron dendritic trees revealed by long-term *in vivo* imaging in neonates

Nakazawa et al.

SUPPLEMENTARY FILES

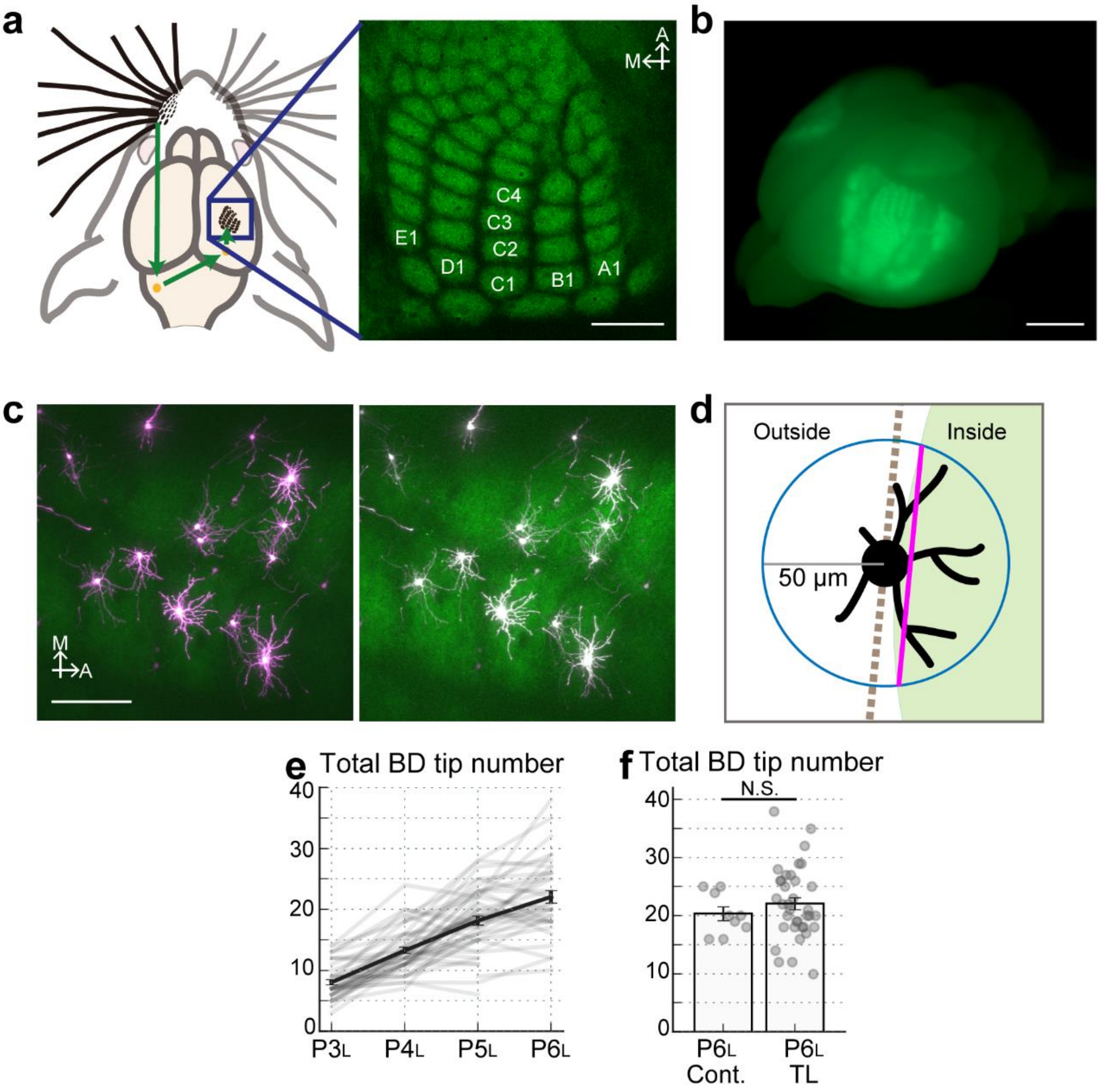

Supplementary Figure 1

Supplementary Figure 1. Barrel as a model system of dendritic structural remodeling.

(a) (Left) A schematic of the barrel map in the mouse somatosensory cortex (barrel cortex), which represents arrangement of whiskers on the face. (Right) The barrel map is visualized by EGFP signals derived from the TCA-GFP Tg mouse. A confocal image of tangential section (100 μm-thick) of barrel cortex L4 of P8 mouse is shown.

(b) The barrel map visualized in the whole brain of P8 TCA-GFP Tg mouse[30].

(c) (Left) A 2-photon image of the barrel cortex L4 of $P6_L$ TCA-GFP mouse transfected with Flpe-based Supernova RFP vector set[29] by *in utero* electroporation at E14. The same image as that of Fig. 1d is shown. TCA termini were visualized by EGFP (green) and a sparse population of L4 neurons were brightly labeled by RFP (magenta). (Right) Green signal was computationally enhanced from the left panel to better show the barrel map.

(d) Inside-Outside boundary (dashed line) was determined as follows. First, a 100 μm-diameter circle (blue circle) that had its center on the cell body is drawn. Second, a line (magenta line) passing through the intersections of the blue circle and TCA cluster boundary (green) is drawn. Then, a line (dashed line) that is parallel to the magenta line and passes through the cell body is the Inside-Outside boundary. Barrel-side half and the opposite-side half of the boundary were defined as “Inside” and “Outside”, respectively.

(e) Increase of L4 neuron BD tip number in TL pups during 3-d-long imaging. Plots of the mean ± SEM (dense) and values of individual neurons (faint) for tip number (n = 51 neurons from 8 mice).

(f) There was no significant difference in total BD tip number between L4 neurons in TL pups (36 neurons from 6 mice) and those in control pups (n = 9 neurons from 2 mice) at $P6_L$ ($p = 0.288$, $g = 0.295$, Welch's t-test). This analysis was done by *in vivo* imaging of TL pups and control pups, to which a cranial window/titanium bar was attached at $P6_L$. Error bars represent SEM.

Scale bars: 500 μm (a), 2 mm (b) and 150 μm (c).

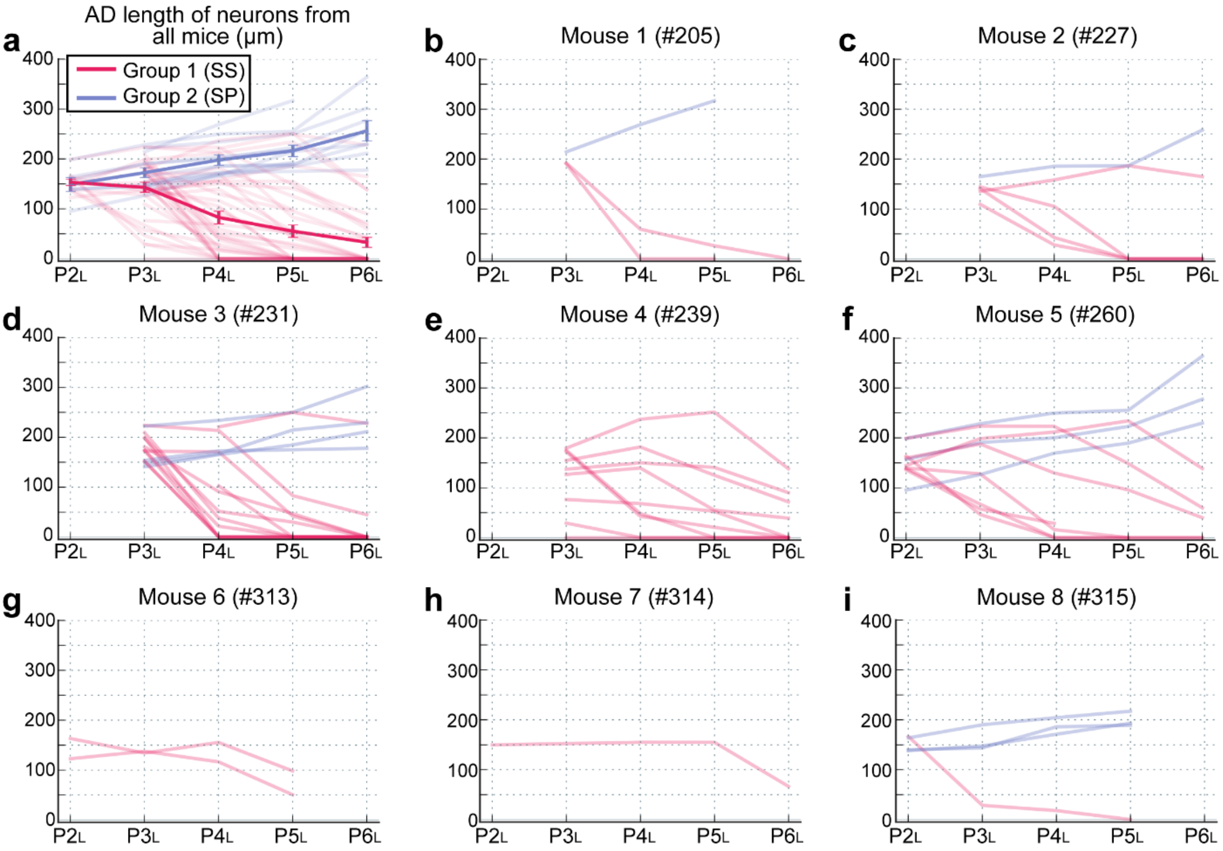

Supplementary Figure 2. Initiation timing and velocity of AD retraction vary among neurons even in the same animal.

(a) Plots of changes of AD length of Group 1 (SS) and Group 2 (SP) neurons between $P2_L$ and $P6_L$ (n = 8 mice). The mean ± SEM (dense) and individual (faint) values are shown. The data between $P3_L$ and $P6_L$ are the same as those of Fig. 2b.

(b–i) The plots of changes of AD length of individual neurons in each mouse. It is intriguingly that the initiation timing and velocity of AD retraction vary among neurons even in the same animals.

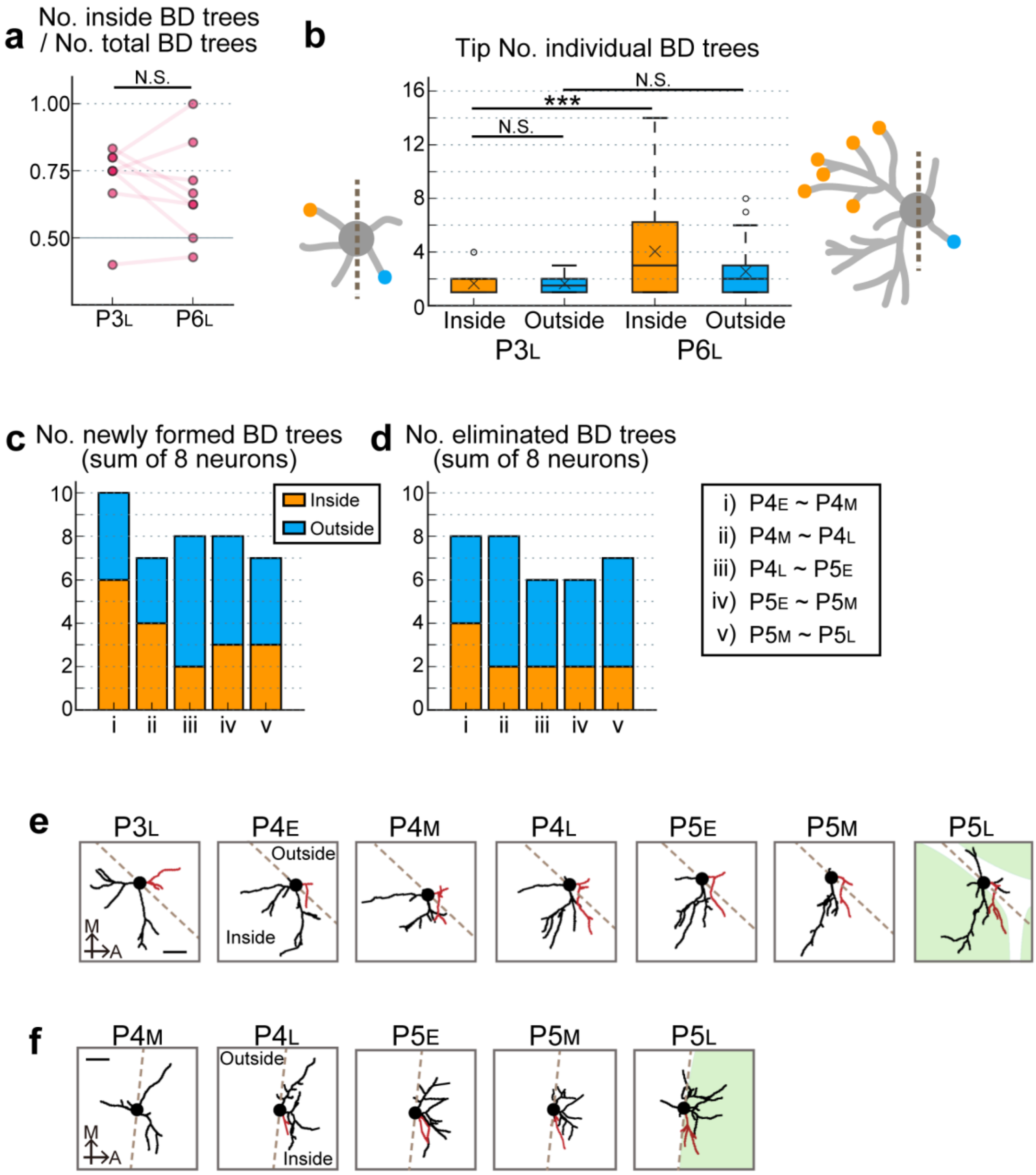

Supplementary Figure 3

Supplementary Figure 3. Aspects of BD tree dynamics of SS neurons.

(a) The ratio of number of inner to total BD trees did not change between $P3_L$ and $P6_L$ ($p = 0.401$, $r = 0.198$, Wilcoxon signed-rank test. $n = 8$ eSS neurons of 4 mice).

(b) The tip number of individual BD trees was similar between inner and outer trees at $P3_L$ ($p = 0.967$, $g = 0.013$, Welch's t-test). The tip number of individual inner trees at $P6_L$ was significantly larger than that at $P3_L$ ($p < 0.001$, $g = 0.939$, Welch's t-test). There was no significant difference between the tip number of individual outer trees at $P3_L$ and that at $P6_L$ ($p = 0.260$, $g = 0.498$, Welch's t-test). Orange dots represent tips of an individual inner tree, and blue dots represent tips of an individual outer tree. p-values were corrected by Holm's correction. Sample sizes are shown in Method. Box plot interpretation is described in the Methods.

(c, d) Formation (c) and elimination (d) of BD trees were observed continuously from $P4_E$ to $P5_L$. Data were collected from the same 8 neurons (4 mice).

(e) An example of outer tree that survived long and was elaborated over time (red). The same neuron as that of Fig. 3a is shown. It should be noted that this outer tree extended its arbors toward the barrel side.

(f) An example of late born winner trees (red). The same neuron as that of Fig. 4a is shown. The winner tree that was first detected at $P4_L$.

Scale bars: 25 μm.

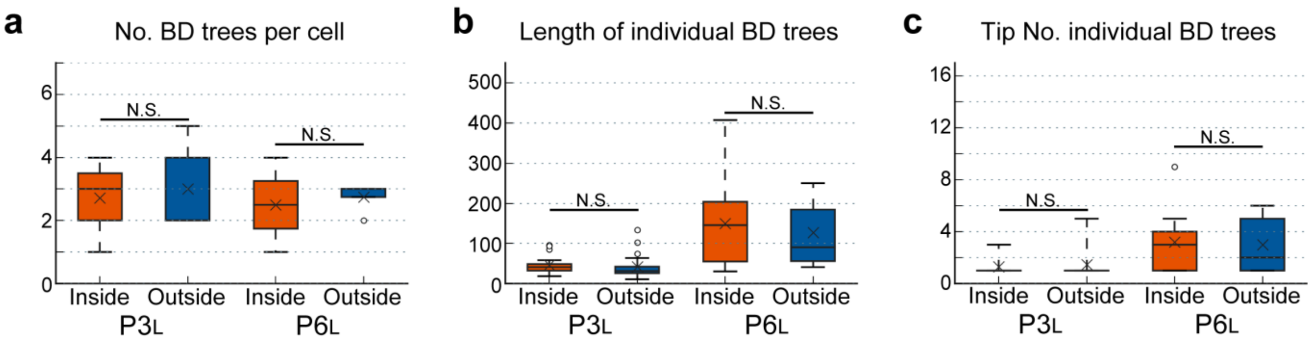

Supplementary Figure 4. Characteristics of eSP neurons.

(a) The numbers of BD trees per cell. $P3_L$ inside vs $P3_L$ outside ($p = 0.856$, $r = 0.049$) and $P6_L$ inside vs $P6_L$ outside ($p = 0.809$, $r = 0.092$). n = 7, 7, 4, 4 neurons. Brunner-Munzel test.

(b) The lengths of individual BD trees. $P3_L$ inside vs $P3_L$ outside ($p = 0.641$, $g = 0.147$) and $P6_L$ inside vs $P6_L$ outside ($p = 0.617$, $g = 0.227$). n = 19, 21, 10, 11 trees. Welch's t-test.

(c) The tip numbers of individual BD trees. $P3_L$ inside vs $P3_L$ outside ($p = 0.572$, $g = 0.177$) and $P6_L$ inside vs $P6_L$ outside ($p = 0.846$, $g = 0.087$). n = 19, 21, 10, 11 trees. Welch's t-test.

Box plot interpretation is described in the Methods.

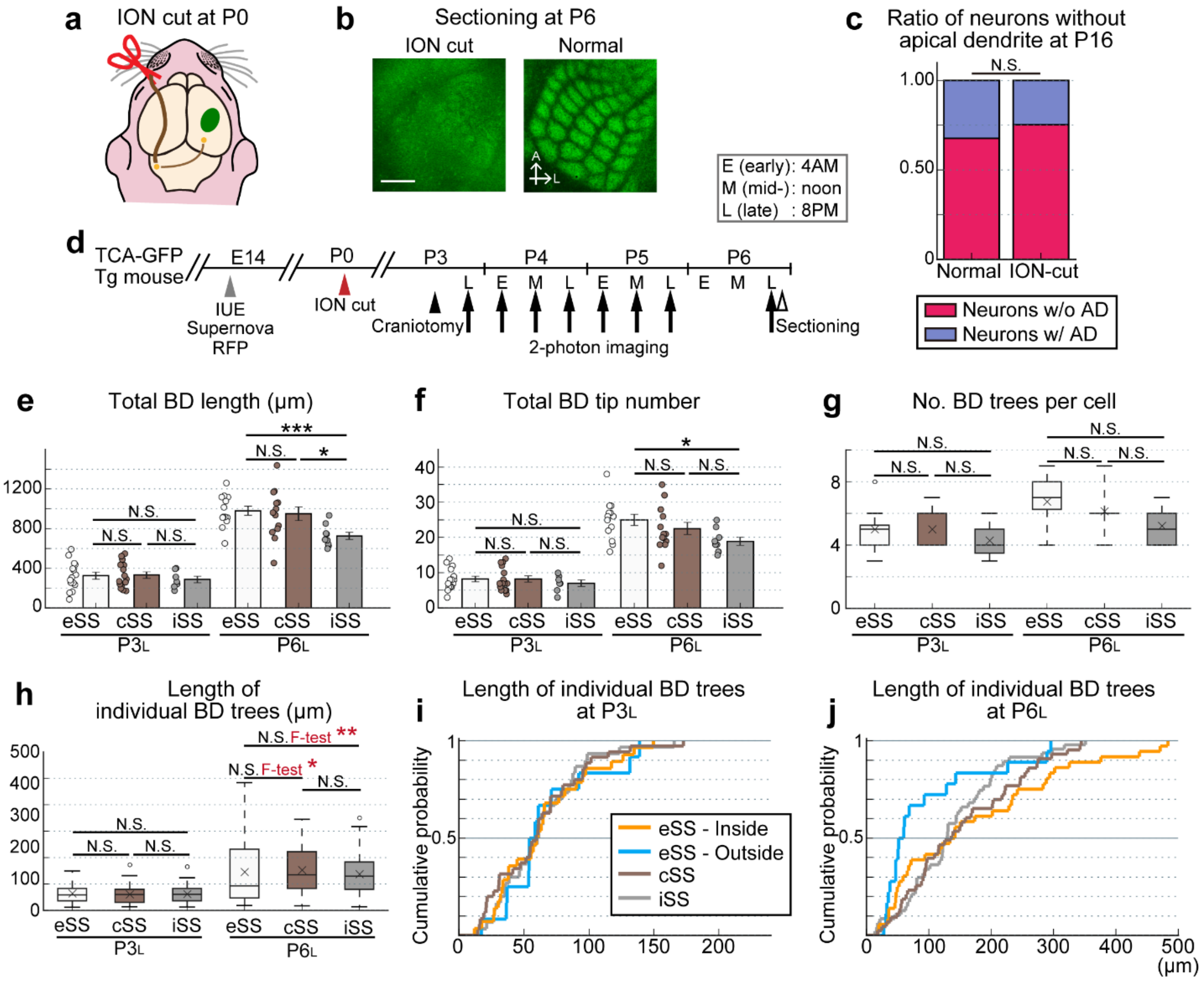

Supplementary Figure 5

Supplementary Figure 5. *In vivo* imaging of Early-ON-cut mice.

(a) IONs were severed at P0 afternoon (Early-ION-cut mice).

(b) Confocal images of tangential slices after *in vivo* imaging at $P6_L$. Barrel maps visualized by EGFP signals of TCA-GFP Tg mouse were impaired in Early-ION-cut mice. Scale bar: 400 μm.

(c) Early-ION-cut (75.3%: 55/73 neurons) and normal (67.8%: 40/59 neurons) mice had similar ratios of neurons without AD (red) at P16 ($p = 0.337$, $\chi^2 = 0.921$, χ^2 test).

(d) Schematic depicting the imaging schedule.

(e) Total BD length of eSS, cSS and iSS neurons at $P3_L$ (eSS vs cSS: $p = 0.894$, $g = 0.049$; eSS vs iSS: $p = 0.914$, $g = 0.291$; cSS vs iSS: $p = 1.071$, $g = 0.375$, Welch's t-test with Holm's correction) and $P6_L$ (eSS vs cSS: $p = 0.735$, $g = 0.135$; eSS vs iSS: $p < 0.001$, $g = 1.767$; cSS vs iSS: $p = 0.020$, $g = 1.100$, Welch's t-test with Holm's correction).

(f) Total BD tip number at $P3_L$ (eSS vs cSS: $p = 1.000$, $g = 0.000$; eSS vs iSS: $p = 1.004$, $g = 0.414$; cSS vs iSS: $p = 0.700$, $g = 0.389$, Welch's t-test with Holm's correction) and $P6_L$ (eSS vs cSS: $p = 0.305$, $g = 0.411$; eSS vs iSS: $p = 0.015$, $g = 1.249$; cSS vs iSS: $p = 0.185$, $g = 0.691$, Welch's t-test with Holm's correction).

(g) Number per cell of BD trees at $P3_L$ (eSS vs cSS: $p = 1.000$, $r = 0.000$; eSS vs iSS: $p = 0.761$, $r = 0.235$; cSS vs iSS: $p = 0.992$, $r = 0.271$, Brunner-Munzel test with Holm's correction) and $P6_L$ (eSS vs cSS: $p = 0.327$, $r = 0.263$; eSS vs iSS: $p = 0.248$, $r = 0.482$; cSS vs iSS: $p = 0.385$, $r = 0.343$, Brunner-Munzel test with Holm's correction).

(h) Length of individual BD trees of eSS, cSS and iSS neurons at $P3_L$, and $P6_L$. At $P3_L$: eSS vs cSS: $p = 1.861$, $g = 0.115$; eSS vs iSS: $p = 1.607$, $g = 0.059$; cSS vs iSS: $p = 0.807$, $g = 0.061$, Welch's t-test with Holm's correction. At $P6_L$: eSS vs cSS: $p = 0.742$, $g = 0.065$; eSS vs iSS: $p = 1.427$, $g = 0.071$; cSS vs iSS: 1.246, $g = 0.174$, Welch's t-test with Holm's correction. See legends of Figs. 5c and 5d for further information, including values of F-test.

(i, j) Cumulative curves of length of individual eSS-Inside, eSS-Outside, cSS and iSS BD trees at $P3_L$ ($n = 28, 12, 35$ and 30 trees) and $P6_L$ ($n = 36, 18, 43$ and 47 trees), respectively. Sample sizes for (e–g) and box plot interpretation are shown in Methods. Error bars: SEM.

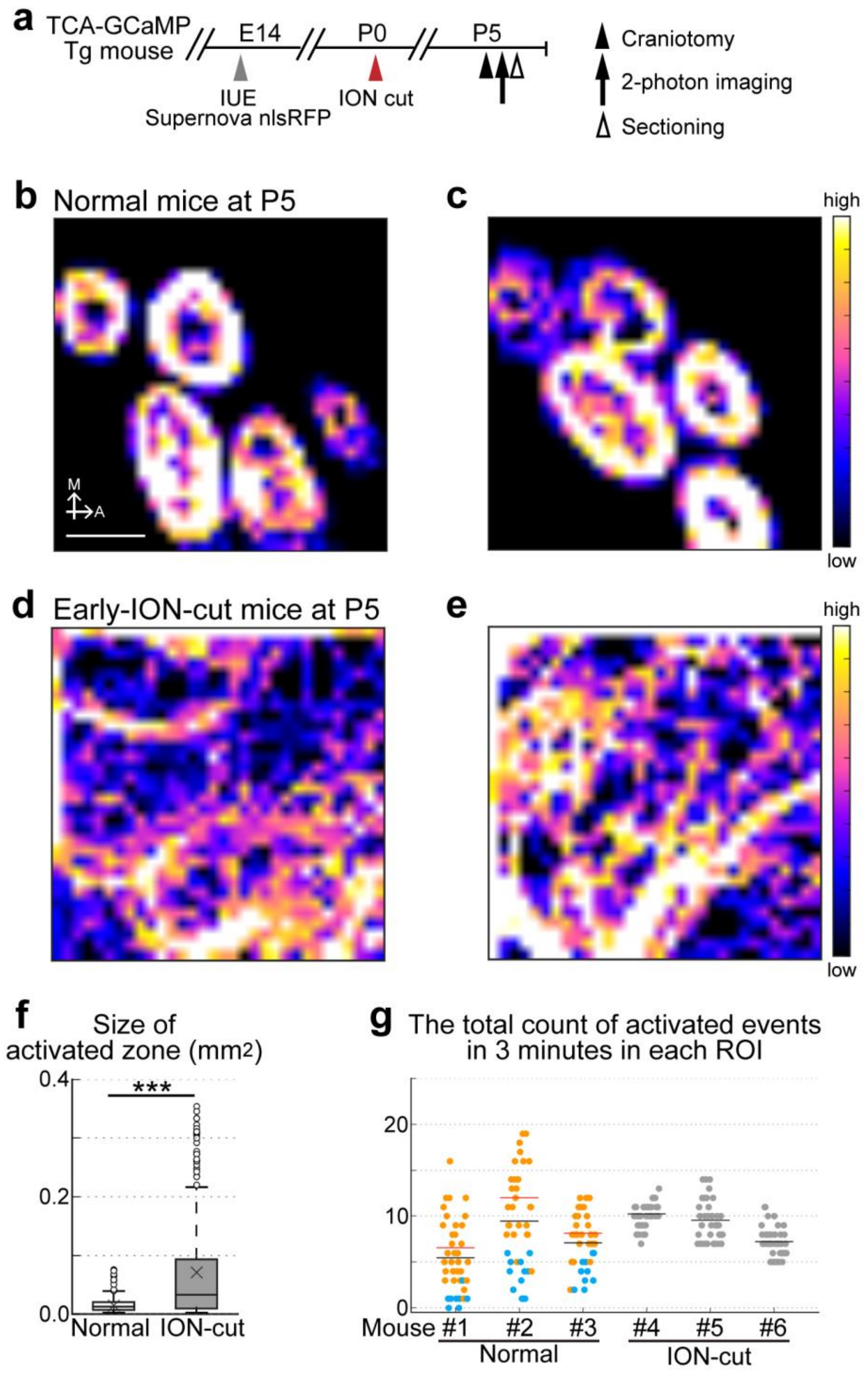

Supplementary Figure 6

Supplementary Figure 6. ION-cut at P0 disrupts patterns of spontaneous activity.

(a) Schematic depicting the experimental schedule.

(b–e) Examples of heat maps of activated zone boundaries observed in the entire imaging sessions (10~15 minutes) in individual mice. Additional two normal (b, c) and two ION-cut (d, e) pups are shown. Scale bar: 150 μm.

(f) Sizes of individual activated zone of normal mice (336 zones in two P5 and one P6 mice) and ION-cut mice (304 zones in three P5 mice) are compared ($p < 0.001$, $g = 0.928$, Welch's t-test).

(g) The total counts of activated events in sequential 3 minutes in each ROI/each mouse. Orange circles: ROIs located on hollows, blue circles: ROIs on septa, gray circles: ROIs on ION-cut mice, black line: average of all ROIs of each mouse, red line: average of ROIs on hollow of each mouse. Hollows and septa were determined manually. Data of Fig.6 are from Mouse #1 and #5. Data of Supplementary Figs. 6b, 6c, 6d and 6e are from Mouse #2, #3, #4 and #6, respectively.

Supplementary Table 1. Summary of neurons which were used in each figure panel.

Neuron			Figure																		
ID (#)	Type[a]	Barrel[b]	2b				2e				2g[c]	2h[c,d]	2j[c]	3b-d, S3a, b [c]	4b-g, S3c, d [c]	S4[c]		5c, d, S5g-j	5e-h	S5e, f	
			$P3_L$	$P4_L$	$P5_L$	$P6_L$	$P3_L$	$P4_L$	$P5_L$	$P6_L$						$P3_L$	$P6_L$			$P3_L$	$P6_L$
205-1	SS	C1	✓	✓	✓	✓	✓	✓	✓	✓	✓	✓	✓	✓				✓		✓	✓
205-3	SS	γ	✓	✓	✓	[g]	✓	✓	✓	[g]										✓	[g]
227-1	SS	C3	✓	✓	✓	✓	✓	✓	✓	✓	✓		✓	✓				✓		✓	✓
227-2	SS	B2	✓	✓	✓	✓	✓	✓	✓	✓	✓	✓	✓	✓				✓		✓	✓
227-3	SS	B1	✓	✓	✓	✓	✓	✓	✓	✓	✓	✓	✓	✓				✓		✓	✓
227-4	SS	C2	✓	✓	✓	✓	✓	✓	✓	✓										✓	✓
231-1	SS	D2	✓	✓	✓	✓	✓	✓	✓	✓	✓	✓	✓	✓	✓			✓	✓	✓	✓
231-2	SS	C2	✓	✓	✓	✓	✓	✓	✓	✓	✓	✓	✓	✓	✓			✓	✓	✓	✓
231-3	SS	C3	✓	✓	✓	✓	✓	✓	✓	✓								✓	✓	✓	✓
231-7	SS	D4	✓	✓	✓	✓	✓	✓	✓	✓								✓	✓	✓	✓
231-8	SS	C4	✓	✓	✓	✓	[g]	✓	✓	✓	[g]		✓		✓[j]				✓[j]	[g]	✓
231-9	SS	B4	[f]	✓	✓	✓	✓	✓	✓	✓										✓	✓
231-10	SS	D5	✓	✓	✓	✓	[g]	✓	✓	✓	[g]		✓		✓[j]				✓[j]	[g]	✓
231-11	SS	D6	✓	✓	✓	✓	[g]	✓	✓	✓	[g]	[g]	✓		✓[j]				✓[j]	[g]	✓
231-12	SS	D6	✓	✓	✓	✓	✓	✓	✓	✓										✓	✓
231-13	SS	D6	✓	✓	✓	✓	[g]	✓	✓	✓	[g]		✓		✓[j]				✓[j]	[g]	✓
231-15	SS	D5	[g]	✓	✓	✓	[g]	✓	✓	✓										[g]	✓
231-16	SS	E7	✓	✓	✓	✓	[g]	✓	✓	✓											
231-17	SS	D7	[g]	✓	✓	✓	[g]	✓	✓	✓											
239-1	SS	C2	✓	✓	✓	✓	✓	✓	✓	✓										✓	✓
239-2	SS	C2	✓	✓	✓	✓	✓	✓	✓	✓	✓		✓	✓	✓			✓	✓	✓	✓
239-3	SS	C3	✓	✓	✓	✓	✓	✓	✓	✓								✓	✓	✓	✓
239-4	SS	D3	✓	✓	✓	✓	✓	✓	✓	✓								✓	✓	✓	✓
239-6	SS	C4	✓	✓	✓	✓	✓	✓	✓	✓	✓	✓	✓	✓	✓			✓	✓	✓	✓
239-7	SS	C4	✓	✓	✓	✓	✓	✓	✓	✓								✓	✓	✓	✓
239-8	SS	D3	✓	✓	✓	✓	[g]	[g]	✓	✓											
239-9	SS	D4	✓	✓	✓	✓	✓	✓	✓	✓								✓	✓	✓	✓
239-10	SS	D4	✓	✓	✓	✓	✓	✓	✓	✓								✓	✓	✓	✓
260-1	SS	B4	✓	✓	✓	✓	✓	✓	✓	✓										✓	✓
260-3	SS	B4	✓	✓	✓	✓	✓	✓	[g]	[g]	✓		[g]							✓	[g]
260-5	SS	C3	✓	✓	[g]	[g]	✓	✓	[g]	[g]	✓		[g]							✓	[g]
260-6	SS	C4	✓	✓	✓	✓	✓	✓	✓	[g]										✓	[g]
260-7	SS	B3	✓	✓	✓	✓	✓	✓	✓	[g]	✓	✓	[g]							✓	[g]

260-9	SS	B2	✓	✓	✓	✓	✓	✓	g)	g)										✓	g)
260-10	SS	B3	✓	✓	✓	g)	✓	✓	✓	g)	✓		g)							✓	g)
313-1	SS	C3	✓	✓	✓	h)	✓	✓	✓	h)	✓	✓	h)							✓	h)
313-2	SS	B2	✓	✓	✓	h)	✓	✓	✓	h)	✓		h)							✓	h)
314-1	SS	C2	i)	✓	✓	✓	i)	✓	✓	✓	i)	i)	✓							i)	✓
315-2	SS	C4	✓	✓	✓	g)	✓	✓	✓	g)	✓		g)							✓	g)
205-2	SP	γ	✓	✓	✓	g)	✓	✓	✓	g)											
227-5	SP	C4	✓	✓	✓	✓	✓	✓	✓	✓	✓	✓	✓			✓	✓				
231-4	SP	D3	✓	✓	✓	✓	✓	✓	✓	✓											
231-5	SP	D3	✓	✓	✓	✓	✓	✓	✓	✓	✓	✓	✓			✓	✓				
231-6	SP	D3	✓	✓	✓	✓	✓	✓	✓	✓	✓	✓	✓			✓	✓				
231-14	SP	D5	✓	✓	✓	✓	g)	✓	✓	✓	g)	g)	✓			g)	✓				
260-2	SP	B4	✓	✓	✓	✓	✓	✓	✓	✓											
260-8	SP	B3	✓	✓	✓	✓	✓	✓	g)	g)	✓	✓	g)			✓	g)				
260-11	SP	C4	✓	✓	✓	✓	✓	✓	✓	✓											
315-1	SP	C4	✓	✓	✓	g)	✓	✓	✓	g)	✓	✓	g)			✓	g)				
315-3	SP	C3	✓	✓	✓	g)	✓	✓	✓	g)	✓	✓	g)			✓	g)				
315-4	SP	C3	✓	✓	✓	g)	✓	✓	✓	g)	✓	✓	g)			✓	g)				
269-2 e)	SS	N/A																✓ j)	✓ j)	g)	✓
269-5 e)	SS	N/A																✓ j)	✓ j)	g)	✓
270-1 e)	SS	N/A																✓	✓	✓	✓
270-2 e)	SS	N/A																✓	✓	✓	✓
270-3 e)	SS	N/A																✓	✓	✓	✓
270-4 e)	SS	N/A																✓	✓	✓	✓
270-6 e)	SS	N/A																✓	✓	✓	✓
356-1 e)	SS	N/A																✓	✓	✓	✓
356-3 e)	SS	N/A																✓	✓	✓	✓

Note:

a) SS: spiny stellate neuron, SP: star pyramid neuron.

b) Barrel column to which the neuron belongs.

c) Neurons located at barrel edge were used (eSS and eSP).

d) eSS with intact AD and eSP were used.

e) ION cut mice.

f) Unanalyzable because AD terminal was out of imaging range.

g) Unanalyzable due to clouded window.

h) Unanalyzable due to death of pup.

i) Imaging was skipped.

j) $P3_L$ was not analyzed due to clouded window.

Supplementary Table 2. Survival-table of newly formed BDs in Figure 4f.

Surviving time-frame	Eliminated outer BDs	n of surviving outer BDs	Eliminated inner BDs	n of survived inner BDs
1	-	22	-	16
2	18	4	5	9
3	1	2	1	7
4	0	1	1	4
5	0	0	0	1
6	-	-	0	1
