# Supplementary material for: Differential dynamics of cortical neuron dendritic trees revealed by long-term in vivo imaging in neonates
Source: Nat Commun. 2018 Aug 6;9:3106. doi: 10.1038/s41467-018-05563-0 (PMC6078955; doi:10.1038/s41467-018-05563-0)
Supplement: Supplementary file 3 — Description of Additional Supplementary Files [file 41467_2018_5563_MOESM3_ESM.pdf]

Description of Additional Supplementary Files

File Name: Supplementary Movie 1
Description: In vivo calcium imaging of the normal mouse at P5 (10-fold speed).

File Name: Supplementary Movie 2
Description: In vivo calcium imaging of the Early-ION-cut mouse at P5 (10-fold speed).
